# Negative regulatory roles of *DE-ETIOLATED1* in flowering time in *Arabidopsis*

**DOI:** 10.1038/srep09728

**Published:** 2015-05-12

**Authors:** Min-Young Kang, Soo-Cheul Yoo, Hye-Young Kwon, Byoung-Doo Lee, Jung-Nam Cho, Yoo-Sun Noh, Nam-Chon Paek

**Affiliations:** 1 Department of Plant Science, Plant Genomics and Breeding Institute, Research Institute of Agriculture and Life Sciences, Seoul National University, Seoul 151-921, Korea; 2 Department of Plant Life and Environmental Science, Hankyong National University, Ansung 456-749, Korea; 3 School of Biological Sciences, Seoul National University, Seoul 151-747, Korea; 4 Crop Biotechnology Institute, GreenBio Science and Technology, Seoul National University, Pyeongchang 232-916, Korea

## Abstract

*Arabidopsis* flowers early under long days (LD) and late under short days (SD). The repressor of photomorphogenesis *DE-ETIOLATED1* (*DET1*) delays flowering; *det1-1* mutants flower early, especially under SD, but the molecular mechanism of DET1 regulation remains unknown. Here we examine the regulatory function of DET1 in repression of flowering. Under SD, the *det1-1* mutation causes daytime expression of *FKF1* and *CO*; however, their altered expression has only a small effect on early flowering in *det1-1* mutants. Notably, DET1 interacts with GI and binding of GI to the *FT* promoter increases in *det1-1* mutants, suggesting that DET1 mainly restricts GI function, directly promoting *FT* expression independent of *CO* expression. Moreover, DET1 interacts with MSI4/FVE, which epigenetically inhibits *FLC* expression, indicating that the lack of *FLC* expression in *det1-1* mutants likely involves altered histone modifications at the *FLC* locus. These data demonstrate that DET1 acts in both photoperiod and autonomous pathways to inhibit expression of *FT* and *SOC1*. Consistent with this, the early flowering of *det1-1* mutants disappears completely in the *ft-1 soc1-2* double mutant background. Thus, we propose that DET1 is a strong repressor of flowering and has a pivotal role in maintaining photoperiod sensitivity in the regulation of flowering time.

The appropriate timing of flowering is tightly linked to the success of reproduction in higher plants. Intrinsic genetic programs and various environmental factors, mainly day length and temperature, determine the transition from vegetative to reproductive development. In particular, photoperiod provides a major cue for controlling flowering time, as perception of light enables plants to synchronize initiation of flowering with seasonal changes in photoperiod^1^.

In *Arabidopsis thaliana*, several signaling components participate in the regulatory circuit promoting photoperiodic flowering, including *GIGANTEA* (*GI*), *CONSTANS* (*CO*), and *FLOWERING LOCUS T* (*FT*)^2^,^3^,^4^. *FT* integrates multiple flowering pathways and FT protein is an essential component of florigen, which moves from the induced leaf to the shoot apex^2^,^5^. CO directly regulates expression of *FT* mRNA and CO mediates between the circadian clock and the control of flowering. CO is stable in the light, but is degraded in the dark by ubiquitin-mediated proteolysis^4^,^6^. GI and FLAVIN-BINDING, KELCH REPEAT, F-BOX PROTEIN 1 (FKF1) form a complex and regulate the timing of *CO* expression. The diurnal expression of *GI* and *FKF1* has little overlap in SD, leading to minimal formation of the GI-FKF1 complex^7^. By contrast, in LD, the more extensive overlap of *GI* and *FKF1* diurnal expression leads to formation of more GI-FKF1 complex. Thus, GI acts as a flowering inducer with FKF1 in the *CO-FT* pathway mainly in LD. In a *CO*-independent flowering pathway, GI can also directly activate *FT* expression by binding to its promoter region^8^, indicating that GI can directly or indirectly induce *FT* transcription in the photoperiod pathway.

In addition to regulation by the photoperiod pathway, genes involved in the autonomous and vernalization pathways also control *FT* expression. *FLOWERING LOCUS C* (*FLC*) has a central place in those two pathways and directly regulates *FT* and *SOC1* expression by binding to their promoters^9^,^10^,^11^. Chromatin remodeling also affects *FLC* expression. For example, MULTICOPY SUPPRESSOR OF IRA1 4 (MSI4)/FVE, in the autonomous pathway, negatively regulates *FLC* expression via histone deacetylation of the *FLC* locus^12^. Furthermore, MSI4/FVE interacts with DDB1 and HDA6, and mediates transcriptional silencing by histone modification of H3K4me3^13^ and H3K27me3^14^. This indicates that MSI4/FVE plays a significant role in *FLC* expression by making a complex with various chromatin remodeling factors.

*DET1*, a repressor of photomorphogenesis, was first identified as a member of the *CONSTITUTIVE PHOTOMORPHOGENIC/DE-ETIOLATED/FUSCA* (*COP/DET/FUS*) gene family^15^. DET1 forms a complex with COP10 and DAMAGED DNA BINDING PROTEIN 1 (DDB1) to promote the activity of ubiquitin-conjugating enzymes (E2) for repression of photomorphogenesis in the ubiquitination pathway^16^,^17^. DET1 also acts as a pacemaker to adjust the period length of the circadian rhythm^18^, possibly through interaction with LHY and CCA1^19^. DET1 acts as a flowering repressor; *det1-1* mutants flower slightly early in LD and extremely early in SD^20^. Despite recent advances in the understanding of DET1 function, the molecular mechanism causing early flowering in *det1-1* mutants remains unknown.

Here we demonstrate that *DET1* delays flowering time in SD, mainly by reducing the affinity of GI binding to the *FT* promoter in the photoperiod pathway. *DET1* also contributes to upregulating *FLC* expression in the autonomous pathway, possibly by weakening the activity of MSI4/FVE in histone modification of the *FLC* locus. These effects, in turn, lead to reduced expression of *FT* and *SOC1*. These findings provide new insights into how DET1 dynamically suppresses flowering in SD and thus plays an important role in maintaining photoperiod sensitivity in *Arabidopsis*.

## Results

### The *det1* mutation alters the expression of flowering-time genes

**Figure 1:**
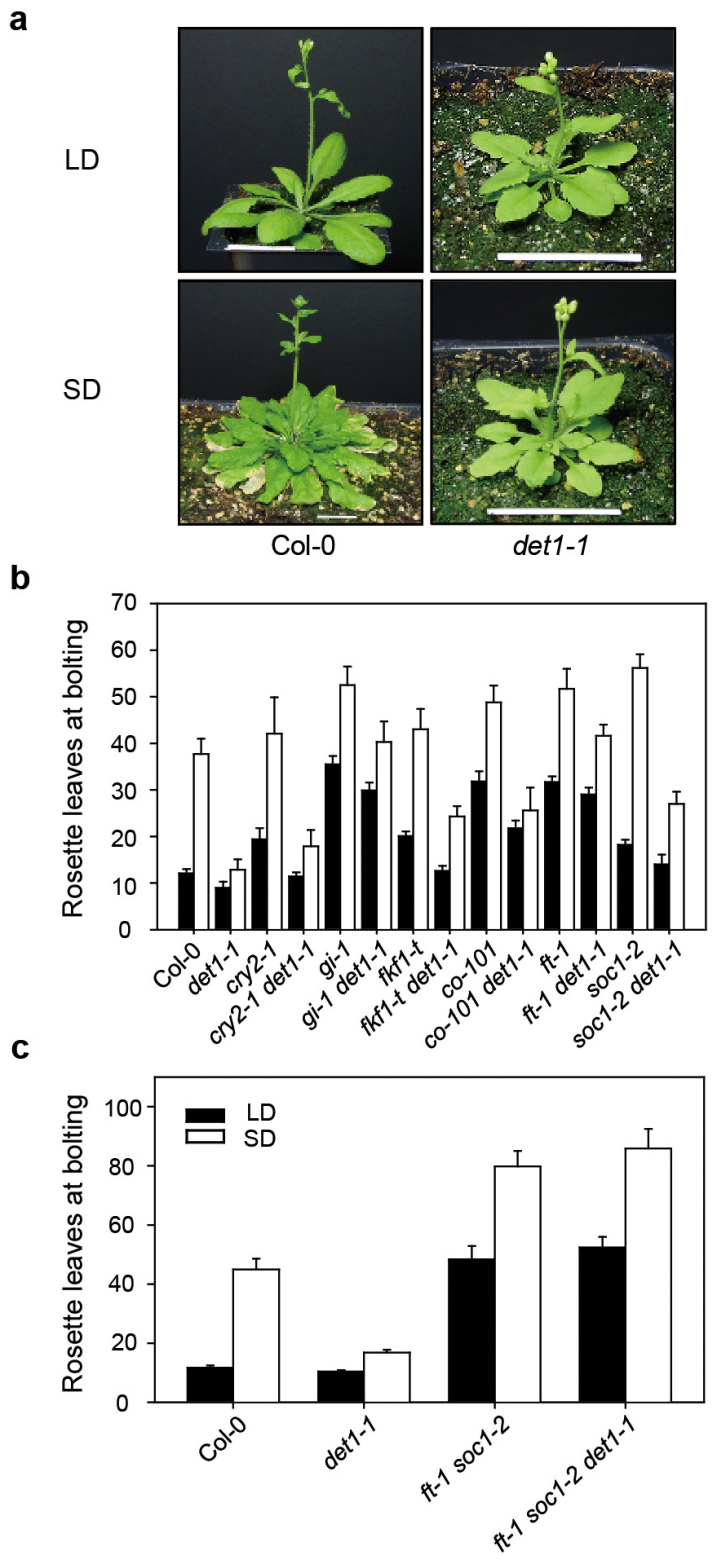
Flowering-time phenotypes of *det1-1* mutants. (a) Phenotypes of wild-type (WT, Col-0 ecotype) and *det1-1* mutant plants. Plants were grown at 22°C under cool-white fluorescent light (90–100 μmol m^−2^s^−1^) in LD (16-h light:8-h dark) or SD (10-h light:14-h dark), and photographed at 2 to 4 days after bolting. Scale bars = 2 cm. (b–c) Genetic analysis to show epistasis between *det1-1* and flowering mutants using double (b) and triple mutants (c). The number of rosette leaves of WT (Col-0) and flowering-time mutants grown under LD (16-h light:8-h dark) and SD (10-h light:14-h dark) in (b), and LD (16-h light:8-h dark) and SD (8-h light:16-h dark) conditions in (c) (see Table S1). Flowering time was measured as the number of rosette leaves at bolting. Means and standard deviations were obtained from more than 20 plants.

The *det1* null mutants are lethal; to study the molecular mechanism by which *DET1* functions in floral repression, we therefore used a weak allele, *det1-1*, and counted the rosette leaf number at bolting to measure flowering time (Fig. 1a, b). We found that *det1-1* mutants flower early under LD and extremely early under SD, which shows that flowering in *det1-1* mutants is photoperiod-insensitive. These results indicate that DET1 acts as a strong floral repressor in SD and has a key role in maintaining the photoperiod sensitivity of the regulation of flowering time in *Arabidopsis*.

**Figure 2:**
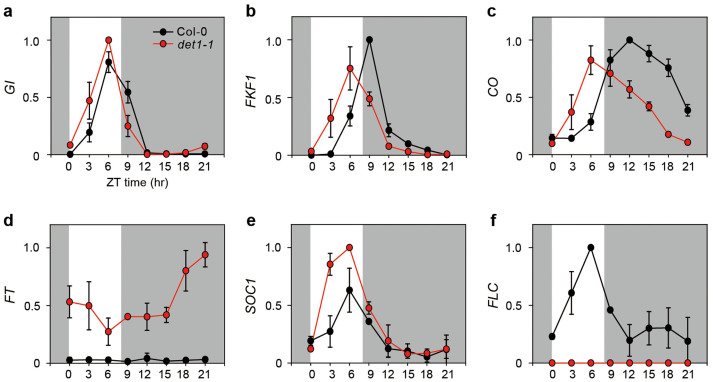
Effect of *det1-1* mutation on *GI*, *FKF1*, *CO*, *FT*, *SOC1*, and *FLC* expression under SD. The expression of *GI* (a), *FKF1* (b), *CO* (c), *FT* (d), *SOC1* (e), and *FLC* (f) was analyzed in Col-0 and *det1-1* mutants by real-time PCR using 3-week-old plants. Plants were grown at 22°C under SD (8-h light:16-h dark) conditions, and plant tissues were harvested every 3 h. *ACT2* expression was used for normalization. Means and standard deviations were obtained from three biological replicates.

The *det1-1* mutation causes period-shortening of clock-regulated gene expression; the internal circadian periods of *CAB2*:*LUC* (encoding a luciferase) expression in *det1-1* mutants were approximately 18 h in continuous darkness and 21 h in continuous light conditions^18^. To investigate whether the circadian defect in *det1-1* mutants causes extremely early flowering under SD (Fig. 1 and Table S1), we analyzed the expression modes of floral inducers by measuring the phases and amplitudes of *GI*, *FKF1*, *CO*, *FT*, and *SOC1* mRNA abundance, in WT and *det1-1* mutants grown in SD (Fig. 2). In WT, *GI* expression peaked at ZT6 (zeitgeber time; 6 h after dawn) during daytime, but the peaks of *FKF1* and *CO* expression occurred at ZT9 and ZT12 during nighttime, respectively, resulting in no *FT* expression^21^. In *det1-1* mutants, *GI*, *FKF1*, *CO*, *FT*, and *SOC1* also showed rhythmic expression (Fig. 2a–e) and *GI* expression did not significantly differ compared with WT (Fig. 2a). However, the peaks of *FKF1* and *CO* expression shifted 3 h and 6 h earlier than those in WT, respectively (Fig. 2b, c). Accordingly, the peaks of *GI*, *FKF1*, and *CO* expression occurred at ZT6 during daytime in *det1-1* mutants under SD. Thus, it appears that the daytime expression of *CO* and light-stabilized CO (Fig. 2c) can activate *FT* expression in *det1-1* mutants under SD (Fig. 2d). The waveform and peak time of *SOC1* expression did not change in *det1-1* mutants, but *SOC1* mRNA abundance increased (Fig. 2e), possibly due to daytime expression of *CO* and/or increased expression of *FT* (Fig. 2c, d)^9^,^22^,^23^. Thus, we first speculated that circadian dysfunction might cause the early flowering in *det1-1* mutants, as previously reported^19^.

**Figure 3:**
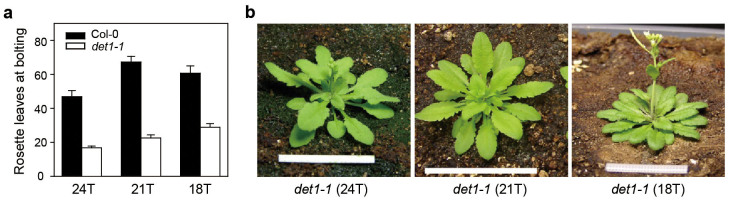
Flowering time of *det1-1* mutants under reduced diurnal cycles. (a) Effect of reduced diurnal cycles on the flowering time of *det1-1* mutants. Plants were entrained in SD (light [L]:dark [D] = 1:2) of 24 h (24 T = 8 L:16 D), 21 h (21 T = 7 L:14 D), and 18 h (18 T = 6 L:12 D). T represents environmental time period. Means and standard deviations were obtained from more than 20 plants. Col-0 means Columbia-0 ecotype (wild type). (b) Phenotypes of *det1-1* mutants after bolting under SD of 24 T, 21 T, and 18 T. Plants were grown at 22–24°C under cool-white fluorescent light (90–100 μmol m^−2^ s^−1^). Scale bars = 2 cm.

To test whether circadian-period shortening causes the extremely early flowering of *det1-1* mutants in SD (Fig. 1 and Table S1), we examined whether the flowering-time defect can be recovered when *det1-1* mutants were entrained in SD (light:dark = 1:2) under reduced diurnal cycles, i.e. environmental time periods (T) of 24 T (8-h light:16-h dark), 21 T (7-h light:14-h dark), and 18 T (6-h light:12-h dark). Although reduced diurnal cycles of 21 T and 18 T slightly delayed flowering compared to normal cycles of 24 T, the *det1-1* mutants still flowered much earlier than WT under SD of 24 T (Fig. 3). To investigate the cause of early flowering in *det1-1* mutants under reduced T cycles, we analyzed the phases and amplitudes of *GI*, *FKF1*, *CO*, *FT*, and *SOC1* mRNA abundance in *det1-1* mutants grown under SD of 18 T (Fig. S1). Unlike the SD of 24 T, the waveforms and peaks of *GI*, *FKF1*, and *CO* expression in *det1-1* mutants were very similar to those of WT. However, *FT* and *SOC1* expression was still upregulated in *det1-1* mutants, suggesting that the internal period-shortening defect in *det1-1* mutants cannot fully explain the extremely early flowering under SD of 24 T. The *FKF1* and *CO* peak shifts likely produce a small effect on early flowering in *det1-1* mutants, because *fkf1-t* and *co-101* mutations delayed flowering in *det1-1* mutants under SD whereas they were almost ineffective in WT^21^ (Fig. 1b and Table S1). Thus, these results strongly suggest that other defects in mechanisms of floral repression lead to photoperiod-insensitive early flowering in *det1-1* mutants, rather than the circadian dysfunction in the *FKF1*-*CO*-*FT* pathway.

### *DET1* mainly functions in the photoperiod and autonomous pathways

To test which genetic pathways of floral induction are responsible for the early flowering phenotype of *det1-1* mutants, we examined the flowering-time phenotypes of double mutants of *det1-1* and mutations with late-flowering phenotypes, specifically *cry2-1*, *fkf1-t*, *gi-1*, *co-101*, *ft-1*, and *soc1-2* (Fig. 1b and Table S1). The *cry2-1* *det1-1* double mutants flowered much earlier than the *cry2-1* single mutants in both LD and SD, suggesting that *DET1* acts downstream of *CRY2*. The *fkf1-t det1-1* and *co-101 det1-1* double mutants exhibited intermediate flowering times compared with *fkf1-t*, *co-101*, and *det1-1* single mutants in both LD and SD, suggesting that although daytime expression of *FKF1* and *CO* contributes to early flowering in SD, *det1-1* mutants can flower early in the absence of FKF1 and CO activity in both photoperiod conditions. In *gi-1* *det1-1* and *ft-1 det1-1* mutants, the early-flowering effect of *det1-1* was almost abolished by *gi-1* or *ft-1* in both LD and SD (Fig. 1b and Table S1), indicating that *GI* and *FT* play major roles in the *DET1*-mediated flowering pathway.

As both the photoperiod and autonomous pathways regulate *SOC1* expression^10^, we further tested whether *DET1* also participates in the autonomous pathway. We found that *soc1-2* *det1-1* double mutants showed intermediate flowering times in both LD and SD. Also, in *ft-1 soc1-2* *det1-1* triple mutants, the early flowering effect of *det1-1* completely disappeared (Fig. 1b, c, and Table S1). These results indicate that the regulation of flowering time by *DET1* does not entirely depend on the *FT*-mediated photoperiod pathway, but also depends on the *SOC1*-mediated autonomous pathway. Thus, we further examined the expression of *FLC*, a major gene in the autonomous pathway, in *det1-1* mutants. We found that the *det1-1* mutants under SD had very low levels of *FLC* mRNA (Fig. 2f), suggesting that DET1 induces *FLC* expression to repress *FT* and *SOC1*. Taking these results together, we concluded that DET1 mainly acts in the photoperiod and autonomous pathways as a strong floral repressor.

### DET1 interacts with GI *in vivo*

**Figure 4:**
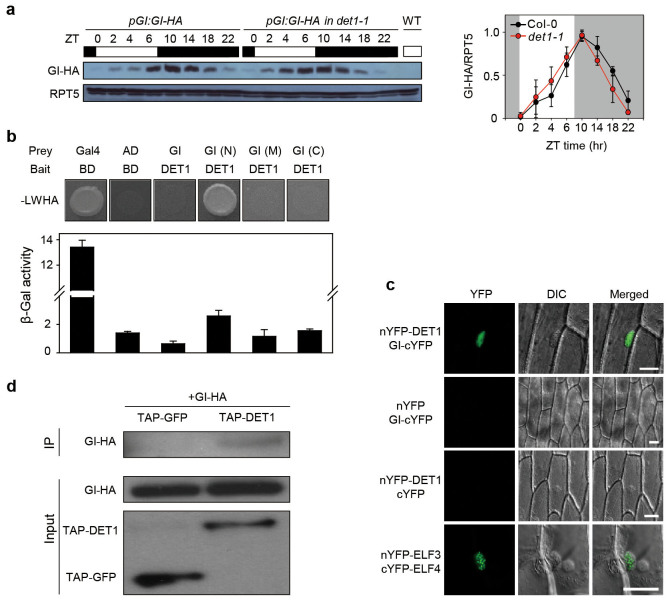
DET1 directly interacts with GI. (a) Comparison of GI protein stability between *pGI:GI-HA* and *pGI:GI-HA* *det1-1* plants under SD conditions. The plant tissues were collected every 2 h during the daytime and every 4 h during the nighttime, using 3-week-old seedlings. GI protein was detected with an anti-HA antibody. RFT5 expression was used for normalization. Means and standard deviations were obtained from three biological replicates. (b) Interaction of DET1-GI was tested by yeast 2-hybrid assay. The bait was full-length DET1. For prey, GI was divided into three pieces: N-terminal (N; 1–507), middle (M; 401–907), and C-terminal (C; 801–1173). Gal4 indicates a positive control. Empty pGBKT7 (BD) and pGADT7 (AD) vectors were used as the negative control. SD medium (-LWHA; lacking tryptophan, leucine, histidine, and adenine) was used to select for the interaction between bait and prey proteins. β-galactosidase (β-Gal) activity assays were performed according to the manufacturer's protocol. Means and standard deviations were obtained from three biological replicates. (c) BiFC analysis of the interaction of between DET1 and GI in the nucleus of an onion epidermal cell. nYFP-ELF3 and cYFP-ELF4 plasmids served as a positive control. For the negative control, empty nYFP/GI-cYFP and nYFP-DET1/cYFP were used. Scale bar = 50 μm. (d) Coimmunoprecipitation of DET1 and GI. Total protein was extracted from 2-week-old seedlings of *p35S:TAP-DET1 pGI:GI-HA gi-2* and *p35S:TAP-GFP pGI:GI-HA gi-2*. IgG beads were used for the pull-down. An anti-HA antibody was used for GI-HA protein band. *p35S:TAP-GFP pGI:GI-HA gi-2* plants served as a negative control. The upper panel is a coimmunoprecipitated sample, and the middle panel is the input sample for GI-HA protein. The lower panel shows input samples of *p35S:TAP-GFP* and *p35S:TAP-DET1*.

GI functions in the photoperiod pathway and *det1-1* mutants did not show significant alterations in *GI* mRNA levels (Fig. 2a), but the *gi-1* mutation nearly abolished the early flowering effect of *det1-1* in *gi-1 det1-1* double mutants (Table S1). Based on these observations, we postulated that DET1 mainly regulates GI at the post-translational level. Thus, we used transgenic plants expressing a tagged GI protein (*pGI:GI-HA gi-2* and *pGI:GI-HA gi-2 det1-1*) to examine whether DET1 negatively regulates GI stability. We found that *det1-1* mutants showed no significant alteration in the rhythmic accumulation of GI protein in SD (Fig. 4a). This indicates that the *det1-1* mutation does not affect GI protein stability.

DET1 interacts with LHY and CCA1, which regulate the circadian rhythms of expression of clock-regulated genes^19^. This raises the possibility that DET1 could negatively regulate GI activity by protein-protein interaction. To examine this, we performed yeast 2-hybrid assays and found that DET1 interacts with the N-terminal region of GI (amino acids [aa] 1-507) (Fig. 4b). To test the *in vivo* interaction of DET1 and GI, we performed bimolecular fluorescence complementation (BiFC) assays. In the onion epidermal cells, we detected reconstituted YFP fluorescence in the nucleus when nYFP-DET1 and GI-cYFP plasmids were co-transformed (Fig. 4c). To further confirm their interaction, we tested whether GI and DET1 co-immunoprecipitate from transgenic plants expressing tagged proteins. To that end, we sampled the *p35S:TAP-DET1 pGI:GI-HA gi-2* and *p35S:TAP-GFP pGI:GI-HA gi-2* (a negative control) transgenic plants at ZT8 in SD, and used antibodies for the TAP tag to immunoprecipitate DET1. We found that HA-GI co-immunoprecipitated with TAP-DET1, but not with TAP-GFP (Fig. 4d). These results indicate that DET1 interacts directly with GI in the nucleus.

### DET1 negatively regulates GI binding to the *FT* promoter

**Figure 5:**
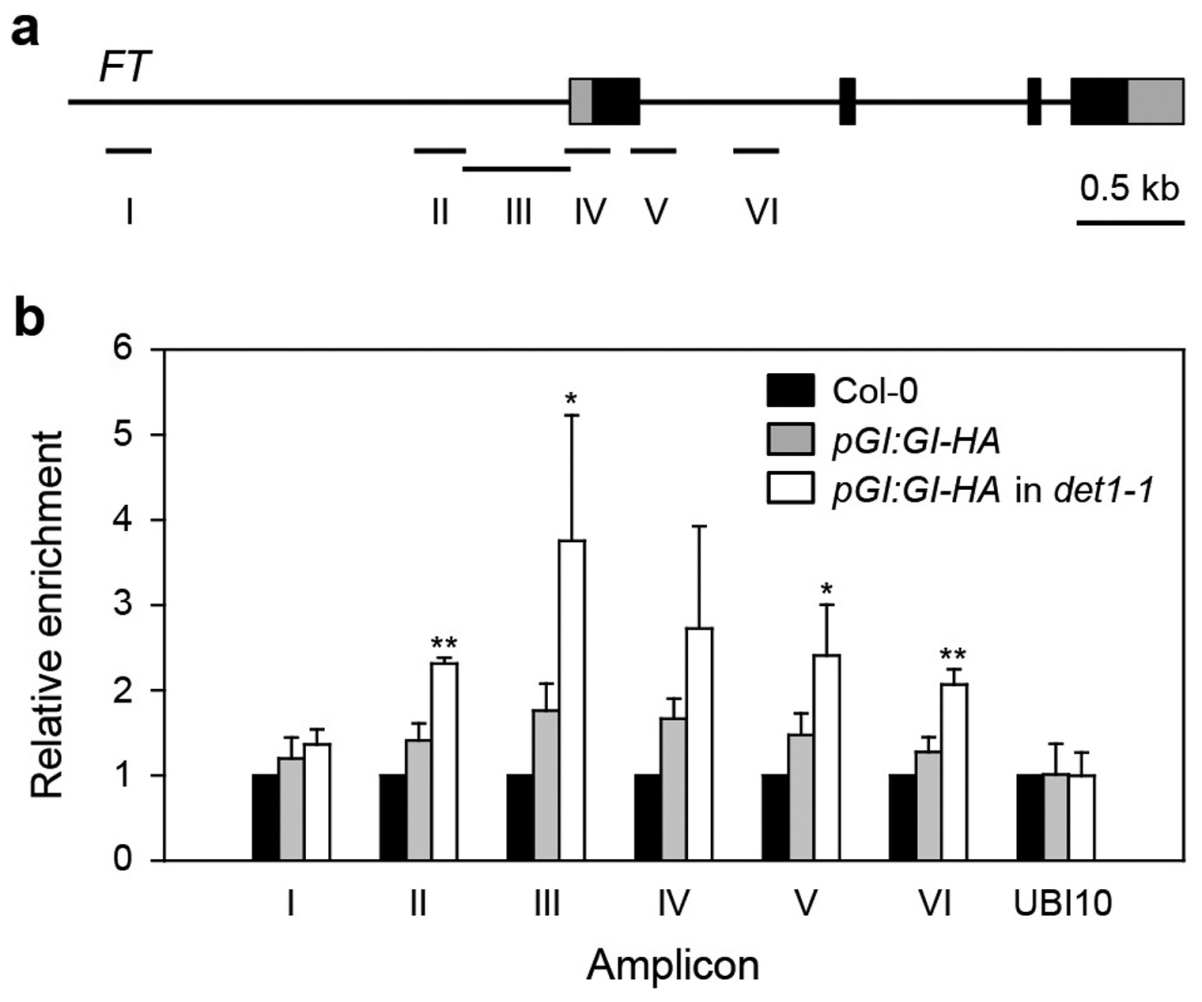
DET1 affects GI binding to the *FT* promoter. (a) Gene structure of *FT* and the amplicon regions for the ChIP assay. Six amplicon locations (I, II, III, IV, V and VI) are shown. (b) *FT* promoter binding affinity of GI in the *det1-1* mutant, relative to the wild type. All samples were harvested at ZT8 under SD (8-h light:16-h dark) conditions. Chromatin isolated from these samples was immunoprecipitated with anti-HA. Relative enrichment in Col-0, *pGI:GI-HA gi-2*, and *pGI:GI-HA gi-2 det1-1* are shown. Means and standard deviations were obtained from three biological replicates. This experiment was replicated at least three times with similar results. *UBIQUITIN 10* (*UBI10*) was used as a negative control. Black, gray, and white boxes represent Col-0, *pGI:GI-HA gi-2*, and *pGI:GI-HA gi-2 det1-1*, respectively. Asterisks indicate statistically significant differences compared to *pGI:GI-HA* as determined by Student's *t*-test (*P < 0.05 and **P < 0.01, respectively).

The *det1-1* mutation does not alter *GI* mRNA expression (Fig. 2a) or GI protein levels (Fig. 4a) but *gi-1* shows nearly complete epistasis to *det1-1* in flowering time (Fig. 1b and Table S1). Based on this observation, we hypothesized that in the photoperiod pathway, DET1 negatively regulates the activity of GI, which directly upregulates *FT* expression through a *CO*-independent pathway^8^. To test whether *det1-1* mutation affects the *GI-FT* module, we performed chromatin immunoprecipitation (ChIP) assays, using *pGI:GI-HA* *gi-2* and *pGI:GI-HA* *gi-2* *det1-1* seedlings entrained in SD, to test whether *det1-1* affects the ability of GI to bind to the *FT* promoter. We collected tissues from 10-day-old seedlings at ZT8 and detected relative enrichment of the promoter regions by PCR with primers for six regions of the *FT* promoter, as described previously^8^. When we compared GI binding affinity to the *FT* promoter regions, the amplicons close to the 5′ untranslated region (UTR) were significantly more enriched in ChIP from *det1-1* mutants (Fig. 5b). This result strongly supports the notion that DET1 plays an important role in the suppression of *FT* transcription by preventing GI binding to the *FT* promoter, and thus contributing to late flowering in SD conditions.

### DET1 positively regulates *FLC* expression to delay flowering time in SD

**Figure 6:**
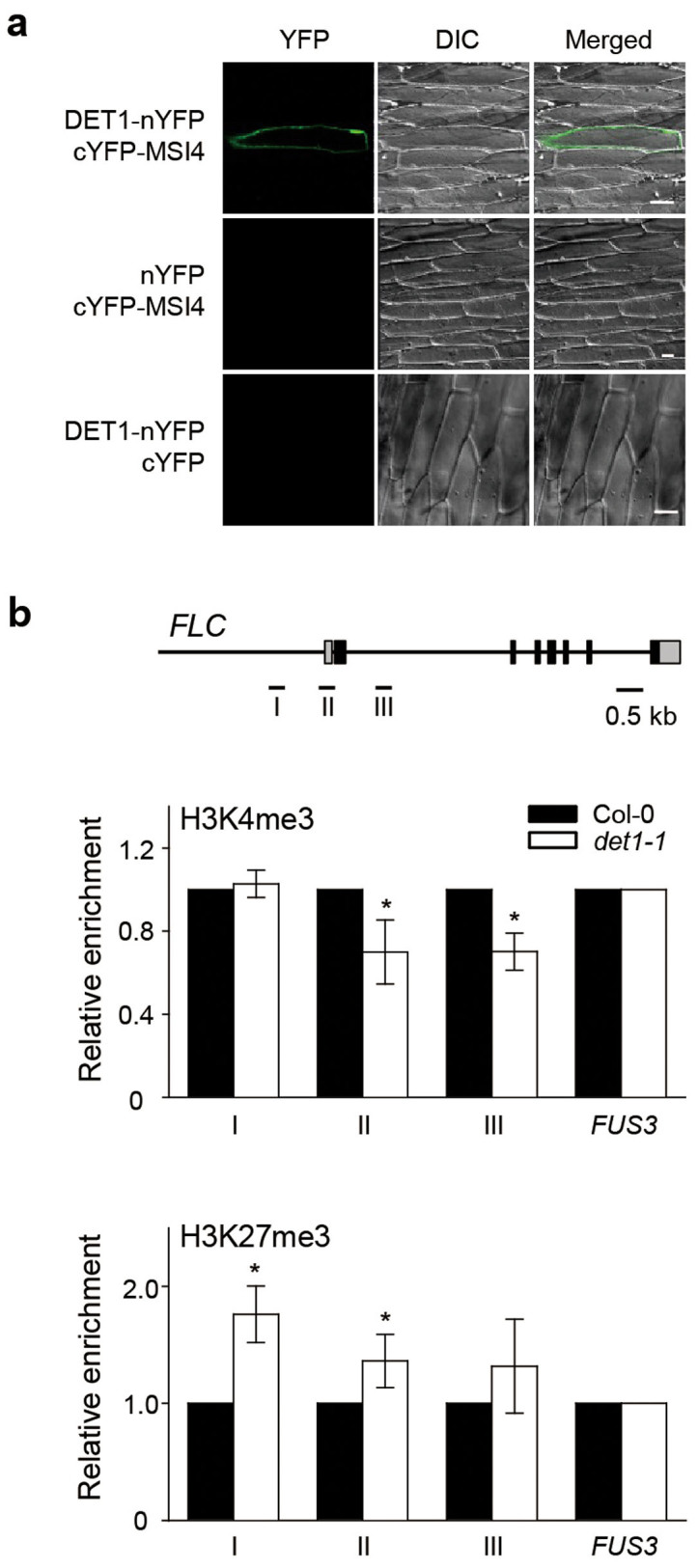
DET1 interacts with MSI4 and regulates histone methylation of the *FLC* locus. (a) BiFC analysis of the interaction between MSI4 and DET1 in onion epidermal cells. For negative controls, nYFP/cYFP-MSI4 and DET1-nYFP/cYFP were used. Scale bar = 50 μm. (b) Relative levels of histone modifications on the *FLC* locus were examined by ChIP analysis using H3K4me3 and H3K27me3 antibodies in Col-0 and *det1-1* plants. The top of the panel represents the *FLC* gene structure and the region used for primers (I, II and III) in the ChIP-quantitative PCR analyses. Chromatin was prepared from 14-day-old seedlings grown under SD (8-h light:16-h dark). *FUSCA 3* (*FUS3*) was used for the normalization of the quantitative PCR analysis. Means and standard deviations were obtained from three biological replicates. This experiment was replicated at least three times with similar results. Asterisks indicate statistically significant difference compared to Col-0 as determined by Student's *t*-test (*P < 0.05).

In the autonomous pathway, *FLC* functions as a key floral repressor and downregulates the transcription of *FT* and *SOC1*^24^,^25^,^26^. As the transcript levels of *FT* and *SOC1* were upregulated in *det1-1* mutants under SD (Fig. 2d, e), and *FLC* expression was almost absent in *det1-1* mutants entrained in SD (Fig. 2f), we reasoned that DET1 also functions to delay flowering in the autonomous pathway by upregulating expression of *FLC*. A previous report showed that the COP10-DET1-DDB1 complex interacts with CUL4^27^ and the DDB1-CUL4 complex interacts with MSI4/FVE to induce *FLC* transcription^14^. Thus, we asked if DET1 interacts with MSI4 to form a DET1-MSI4 complex to regulate *FLC* mRNA levels. To test this, we examined the *in vivo* interaction of MSI4-DET1 by BiFC assays (Fig. 6a). We detected strong YFP fluorescence in the nuclei of cells co-transformed with plasmids expressing DET1-nYFP and cYFP-MSI4, indicating that DET1 interacts with MSI4, which directly binds to the *FLC* promoter to repress *FLC* transcription.

Since MSI4 binds to the *FLC* promoter and alters histone modification, specifically H3K27me3 and H3K4me3, at the *FLC* locus^13^,^14^, we further examined the histone methylation levels of the *FLC* locus, using anti-H3K27me3 and anti-H3K4me3 antibodies in WT and *det1-1* mutants. The ChIP analysis revealed that *det1-1* mutants maintained higher levels of H3K27me3 and lower levels of H3K4me3 at the *FLC* locus than did WT (Fig. 6b), consistent with the histone modification states observed in the early-flowering *hos1-3* mutants^28^. Taking these results together, we suggest that the DET1-MSI4/FVE complex likely contributes to late flowering in SD by altering histone modification of the *FLC* locus in the autonomous pathway.

## Discussion

**Figure 7:**
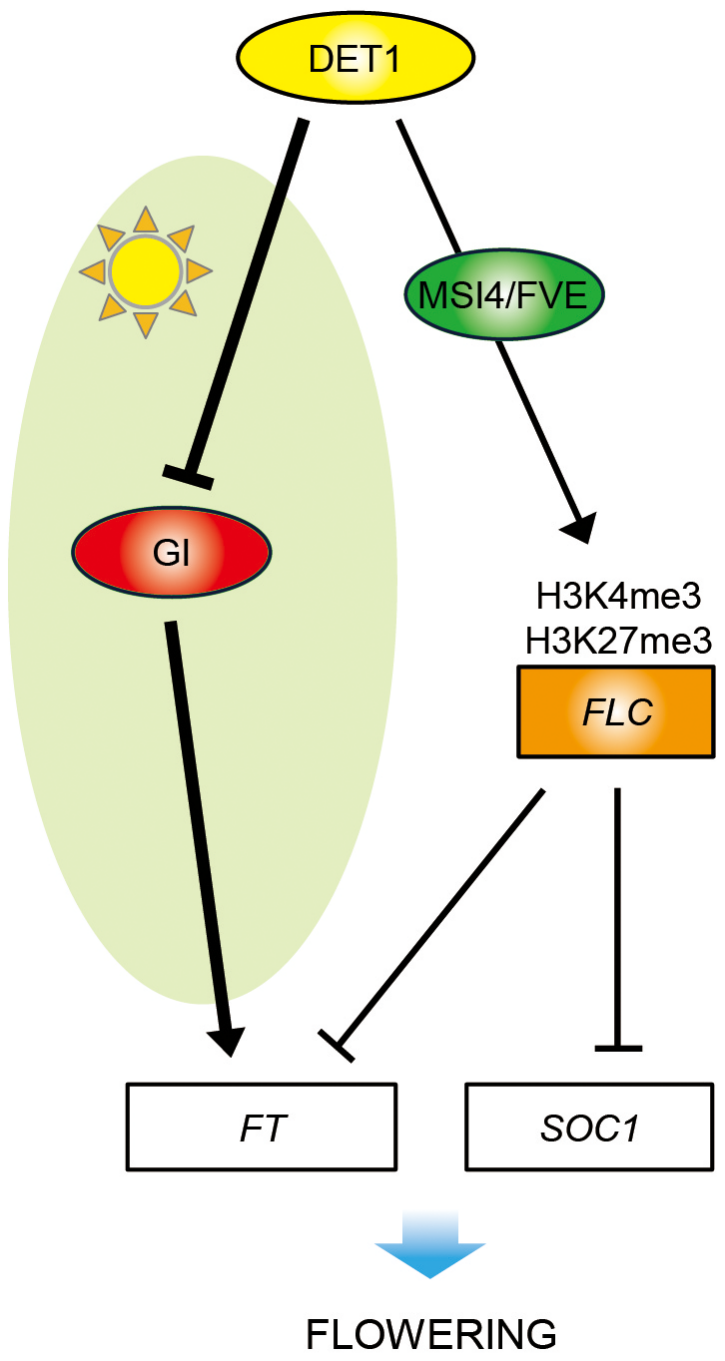
Working model of DET1 function in floral repression in *Arabidopsis*. DET1 suppresses *FT* and *SOC1* expression through the photoperiod and autonomous pathways of flowering. In the photoperiod pathway, DET1 mainly represses flowering by modulating GI-mediated floral induction at the transcriptional and post-translational levels during daytime under SD. DET1 represses the function of daytime-expressed GI by preventing GI from binding to the *FT* promoter in a *CO*-independent pathway. In the autonomous pathway, DET1 interacts with MSI4/FVE and possibly modulates trimethylation of *FLC* chromatin to epigenetically induce *FLC* expression. Genes and proteins are represented as rectangles and ovals, respectively.

DET1 is involved in repression of photomorphogenesis in the ubiquitination pathway^16^,^17^,^29^, light-response regulatory pathway^20^, and circadian period^18^,^19^. However, the function of DET1 in the regulation of flowering time remains unclear. In this study, we provide evidence showing how DET1 regulates photoperiod sensitivity by delaying flowering time in SD. For example, *det1-1* mutants showed increased GI activity (Fig. 5) and epigenetic silencing of *FLC* expression (Fig. 6), resulting in upregulation of *FT* and *SOC1*. Thus, we propose a model for the regulatory role of DET1 in both photoperiod and autonomous pathways (Fig. 7).

In this study, we showed that *gi-1* and *ft-1* nearly completely suppressed the early flowering of *det1-1* mutants and that DET1 directly interacts with GI *in vitro* and *in vivo* (Fig. 4). However, DET1 does not interact with the light-input components PHYA, PHYB, CRY1 C-terminus (CCT1), or CRY2 C-terminus (CCT2), or the floral inducers CO or FKF1 (Fig. S3), indicating that DET1 has a unique role in the posttranslational regulation of GI activity in the photoperiod pathway. A previous study revealed that EARLY FLOWERING4 (ELF4), one of the circadian-clock components^30^, acts upstream of GI^31^. ELF4 represses GI binding to the *CO* promoter to control flowering^32^. Our results revealed that *co-101 det1-1* mutants showed intermediate flowering-time phenotypes, but in *ft-1 det1-1* mutants, the early flowering phenotype of *det1-1* almost completely disappeared under LD (Fig. 1b), indicating that DET1 function in the regulation of photoperiodic flowering mainly depends on *FT* expression. Thus, we hypothesized that DET1 regulates GI binding to the *FT* promoter to delay flowering time and showed that GI binding to the *FT* promoter significantly increased in the *det1-1* mutant background (Fig. 5). This result indicates that DET1 represses *FT* expression via direct regulation of GI binding to the *FT* promoter.

DET1 functions as a repressor of photomorphogenesis in darkness by forming a complex with COP10 and DDB1 and promoting the activity of ubiquitin-conjugating E2 enzymes in the ubiquitination pathway^16^,^17^. The RING-type E3 ubiquitin ligase COP1, a member of the COP/DET/FUS family^15^, also represses photomorphogenesis in darkness; *cop1-4* mutants display very similar phenotypes to *det1-1* mutants, such as short hypocotyls and opened cotyledons^34^. This implies a potential functional connection between DET1 and COP1. Indeed, COP1 interacts with COP10, but not with DET1^16^, suggesting that COP1 could interact with the COP10-DET1-DDB1 (CDD) complex to repress photomorphogenesis. In addition, *cop1-4* mutants flower extremely early under SD, similar to *det1-1* mutants^33^. Thus, the CDD complex may function with COP1 in regulation of flowering time, although we have no direct evidence because the *det1-1 cop1-4* double mutant is lethal^34^. COP1 directly controls GI stability by interacting with GI in the presence of ELF3 for photoperiodic flowering^33^. However, DET1 does not regulate GI stability but does negatively affect GI binding to the *FT* promoter (Fig. 4a). Therefore, although DET1 and COP1 have very similar mutant phenotypes and post-translational behavior, they seem to regulate GI function independently through distinct molecular mechanisms.

Other negative regulators of *FT* transcription, including FLC, SVP, TEM1, and TEM2, bind to the regions near the 5′UTR of *FT*. In single mutants of these regulatory genes, *FT* mRNA expression increases to levels similar to those seen in *det1-1* mutants^11^,^35^,^36^. Notably, SVP, TEM1, and TEM2 interact with GI to regulate *FT* expression, although the regulatory function of their interaction is not clearly understood^8^. Therefore, DET1 could be involved in the function of these *FT* repressors. To investigate this possibility, we examined the interaction of DET1 with these four *FT* repressors by yeast 2-hybrid assays, which revealed that DET1 does not interact with FLC, SVP, TEM1, or TEM2 (Fig. S4). This result strongly suggests that DET1 may regulate the *GI-FT* module independent of these known *FT* repressors.

In addition, we revealed that DET1 regulates the expression of *FLC*, a key component in the autonomous pathway. We found that the *det1-1* mutants showed a remarkable decrease in *FLC* mRNA levels and had altered levels of H3K4me3 and H3K27me3 (Figs. 2f and 6b), as observed in the early-flowering *hos1-3* mutants^28^. Furthermore, our examination of the components of the CDD complex showed that in addition to interacting with DDB1, DET1 also interacts with MSI4/FVE, which repress *FLC* expression in the autonomous pathway (Fig. 6a)^14^. This indicates that DET1 represses *FLC* expression possibly through direct interaction with MSI4/FVE. Meanwhile, FLC negatively regulates not only *FT* but also the downstream factor *SOC1*, which encodes a MADS box transcription factor^37^. In genetic analysis, *ft-1* was completely epistatic to *det1-1* in LD, but in SD the *ft-1* *det1-1* double mutants showed an intermediate phenotype, indicating incomplete epistasis. Consistent with this, *SOC1* expression was upregulated in *det1-1* mutants (Fig. 2e), but *soc1-2* did not rescue the early flowering of *det1-1* (Fig. 1b and Table S1). Notably, the *ft-1 soc1-2 det1-1* triple mutants showed complete suppression of the early flowering of *det1-1* in both photoperiods. This supports the idea that DET1 suppresses both *FT* and *SOC1* via promoting *FLC* expression in the autonomous pathway.

DET1 interacts with LHY/CCA1 and is required for transcriptional repression of CCA1/LHY target genes such as *TOC1*^19^. These observations indicate that DET1 functions with LHY/CCA1 to regulate the circadian rhythms of evening genes. Moreover, DET1 could act with LHY/CCA1 to negatively regulate GI binding to the *FT* promoter mainly in SD, because *lhy cca1* double mutants also exhibit photoperiod-insensitive early flowering^38^. To prove this hypothesis will require further analysis, such as examination of the *in vivo* interaction of CCA1-GI or LHY-GI, and GI binding activity to the *FT* promoter in either *lhy cca1* double mutants or *LHY* or *CCA1* overexpressors.

Based on these data, we propose a model for the molecular mechanism by which DET1 represses flowering in non-inductive SD conditions (Fig. 7). In WT plants, the absence of *FT* expression under SD can be explained by the incongruity of peak expression of *FKF1* and *GI*; *GI* peaks in the late afternoon but *FKF1* peaks at night, leading to reduced expression of *CO* and *FT* during daytime^21^. As GI also directly induces *FT* expression in a *CO*-independent pathway^8^, we wondered why *GI*, which is expressed in the afternoon^21^ (Fig. 2a), is not capable of inducing *FT* expression under SD (Fig. 2a, d). In this study, we found that DET1 suppresses *FT* transcription by repressing GI binding activity to the *FT* promoter (Fig. 5b). This model is further supported by genetic analysis showing that *gi-1* and *ft-1* are almost completely epistatic to *det1-1* (Fig. 1b and Table S1), indicating that DET1 mainly regulates flowering via GI.

In conclusion, we propose that DET1 functions as a strong repressor of flowering, acting in both photoperiodic and autonomous pathways (Fig. 7); DET1 suppresses flowering mainly by decreasing GI binding activity to the *FT* promoter in the photoperiod pathway and epigenetically upregulating *FLC* expression in the autonomous pathway. Whether DET1 acts in the CDD complex^17^ to delay flowering time under SD in *Arabidopsis* remains to be elucidated.

## Methods

### Plant materials and growth conditions

All the *Arabidopsis thaliana* lines used in this study are in the Columbia (Col-0) genetic background. Flowering-time mutants were obtained from the Arabidopsis Biological Resource Center (USA), except for *det1-1* which was kindly provided by Joanne Chory. *cry2-1* (CS3732), *gi-1* (CS3123), *soc1-2* and *ft-1*^39^, *fkf1-t*^40^, and *co-101*^41^ were used for genetic analysis. To create double and triple mutants, F_1_ heterozygotes were obtained by crossing the *det1-1* mutant as the female plant with other flowering-time mutants as pollen donors. To select correct transformants, the plants showing the *det1-1* morphological phenotype were first isolated from F_3_ plants, and flowering-time mutations were finally confirmed by PCR-based genotyping. Plants were grown on soil at a constant 22°C under white fluorescent light (90-100 μmol m^−2^s^−1^) in LD (16 h light:8 h dark) and SD (10 h light:14 h dark) or SD (8 h light:16 h dark).

### Analysis of flowering time

The bolting date was measured as the number of days from seed sowing to opening of the first flower and as the total number of rosette leaves at bolting. Data were obtained from three experimental replications (20 to 60 plants per replication).

### RNA preparation and quantitative real-time PCR analysis

Tissue samples were collected every 3 h from 3-week-old seedlings. Total RNA was extracted with the plant RNA extraction kit (Macrogen). For each sample, 2 μg of total mRNA was reverse transcribed using M-MLV reverse transcriptase (Promega). The level of the transcripts was measured by real-time PCR, using GoTaq qPCR Master Mix (Promega) and the Light Cycler 2.0 instrument (Roche). Each PCR was repeated at least three times using biologically independent samples. The amount of each RNA level was determined using specific primers. The primers used for real-time PCR are listed in Table S2.

### Yeast 2-hybrid assays

The full-length cDNAs of *DET1, GI, PHYA, PHYB, CCT1, CCT2, CO, FKF1, FLC, SVP, TEM1,* and *TEM2* were amplified from wild-type total RNA using RT-PCR. GI was divided into three parts: GI N-terminal (aa 1-507), GI middle (aa 401-907), and GI C-terminal (aa 801-1173) regions. The PCR products were cloned into pGBKT7 and pGADT7 vectors (MATCHMAKER GAL4 TWO-hybrid system 3, Clontech) to get the bait and prey clones. For the interaction study, plasmids containing fusion proteins were transformed into *Saccharomyces cerevisiae* AH109 and grown on media lacking adenine, leucine, histidine, and tryptophan. Galactosidase activity assays were performed according to the manufacturer's protocol.

### *In vivo* pull-down assays

*TAP-DET1* and *TAP-GFP* were from Xing Wang Deng. *pGI:GI-HA gi-2 det1-1* was obtained by crossing *pGI:GI-H*A *gi-2* and *det1-1*. For DET1-GI binding assays, *TAP-DET pGI:GI-HA gi-2* and *TAP-GFP pGI:GI-HA gi-2* plants were grown on MS medium in SD (8 h light:16 h dark) for 10 days and then vacuum infiltrated for 7 ~ 10 min in 1X MS (Duchefa) liquid medium supplemented with 50 mM MG132 (Sigma) for proteasome inhibitor treatment. After that, plants were incubated for 10 h under light conditions. These plants were homogenized and total proteins were extracted in total protein extract buffer [50 mM Tris-HCl (pH 7.5), 100 mM NaCl, 10 mM MgCl_2_, 1 mM EDTA (pH 8.0), 10% glycerol, 1 mM PMSF, 1 mM DTT]. These experiments were performed with IgG beads for TAP-IP. After washing, the immunoprecipitated fractions were analyzed by immunoblotting. The TAP-DET1 and GI fusion proteins were detected by using anti-HA antibody.

### Bimolecular fluorescence complementation assays

Each cDNA of *GI, ELF3, DET1, and MSI4* was cloned into the BiFC gateway vectors^42^ to examine their *in vivo* interactions. For partial YFP-tagged DET1, and MSI4 constructs, the cDNA of the gene was obtained by RT-PCR from wild-type (WT, Col-0) plants and fused into four BiFC plasmid sets, pSAT5-DEST-cEYFP(175-end)-C1(B) (pE3130), pSAT5(A)-DEST-cEYFP(175-end)-N1 (pE3132), pSAT4(A)-DEST-nEYFP(1-174)-N1(pE3134), and pSAT4-DEST-nEYFP(1-174)-C1 (pE3136). Partial YFP-tagged ELF3 and GI constructs were previously described^33^. Each pair of recombinant plasmids encoding nEYFP and cEYFP fusions was mixed 1:1 (w/w), co-bombarded into onion epidermal layers using a DNA particle delivery system (Biolistic PDS-1000/He, BioRad), and incubated on MS solid media with MG132 (50 mM) for 16–24 h at 22°C under light or dark incubation, followed by observation and image analysis using a confocal laser scanning microscope (Carl Zeiss LSM710).

### Chromatin immunoprecipitation assay

For the ChIP assay, Col-0, *pGI:GI-HA gi-2*, and *pGI:GI-HA gi-2 det1-1* plants were grown for 10 days under SD (8 h light:16 h dark) conditions and collected at ZT8. The samples were cross-linked with 1% formaldehyde, ground to powder in liquid nitrogen, and then sonicated^43^. The sonicated chromatin complexes were bound with anti-HA antibody (ab9110, Abcam) for immunoprecipitation. The amount of DNA fragment was analyzed by quantitative real-time PCR (qPCR) using specific primers. *UBI10* was used as an internal standard for normalization. The primers used for qPCR are listed in Table S2. For another ChIP assay, Col-0 and *det1-1* plants were grown for 14 days under SD (8 h light/16 h dark) conditions and collected at ZT8. For immunoprecipitation, we used the anti-trimethyl H3K4 (07-473, Millipore), and anti-trimethyl H3K27 (07-449, Millipore). *FUS3* was used as an internal standard for normalization^14^. Experiments were performed with three biological repeats.

## Author Contributions

M.-Y.K., S.-C.Y., Y.-S.N. and N.-C.P. conceived the study and designed the research. M.-Y.K., H.-Y.K., J.-N.C. and B.-D.L. performed experiments. M.-Y.K. and S.-C.Y. analyzed data with suggestions by Y.-S.N. and N.-C.P. M.-Y.K., S.-C.Y. and N.-C.P. wrote the article. All authors read and approved the final manuscript.

## Supplementary Material

Supplementary Information Supplementary Information
