# Supplementary material for: Negative regulatory roles of DE-ETIOLATED1 in flowering time in Arabidopsis
Source: Sci Rep. 2015 May 12;5:9728. doi: 10.1038/srep09728 (PMC4428065; doi:10.1038/srep09728)
Supplement: Supplementary Information [file srep09728-s1.pdf]

Supplementary Information

Negative regulatory roles of *DE-ETIOLATED1* in flowering time in *Arabidopsis*

Min-Young Kang, Soo-Cheul Yoo, Hye-Young Kwon, Byoung-Doo Lee, Jung-Nam Cho, Yoo-Sun Noh & Nam-Chon Paek

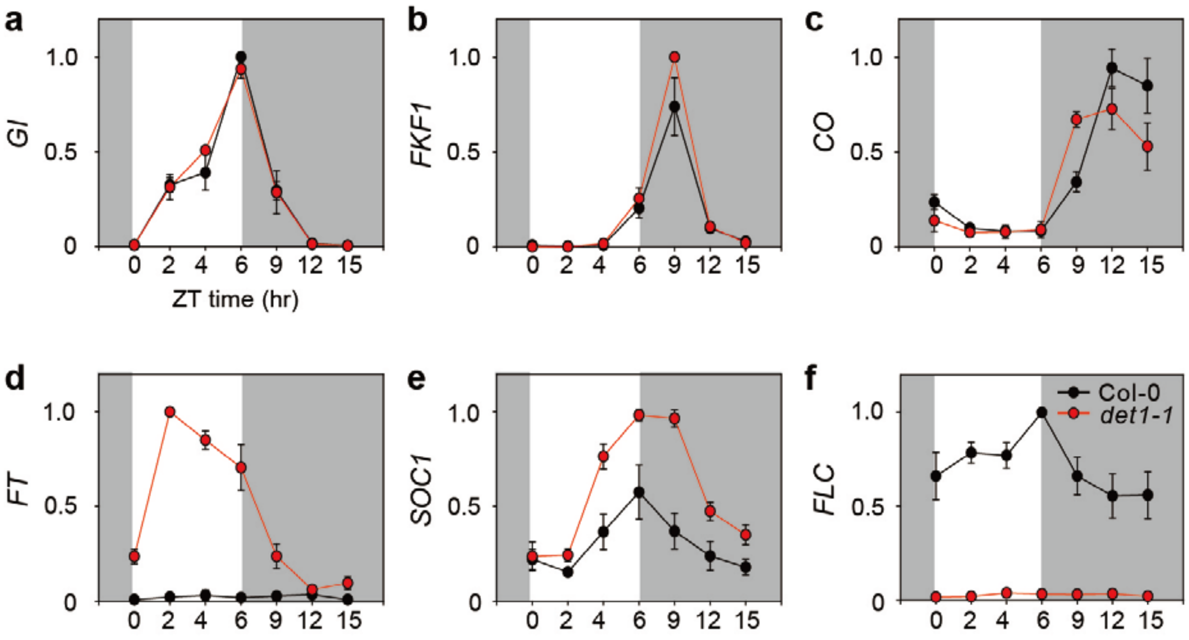

Figure S1 | Effect of *det1-1* mutation on *GI*, *FKF1*, *CO*, *FT*, *SOC1*, and *FLC* expression under 18T (6-h light:12-h dark). (**a-f**) The expression of *GI* (**a**), *FKF1* (**b**), *CO* (**c**), *FT* (**d**), *SOC1* (**e**), and *FLC* (**f**) genes was analyzed in Col-0 and *det1-1* mutants by real-time PCR using 10-day-old plants. Plants were grown at 22°C under SD (6-h light:12-h dark) conditions, and plant tissues were harvested every 2 h during daytime and every 3 h during nighttime. *ACT2* expression was used for normalization. Means and standard deviations were obtained from three biological replicates.

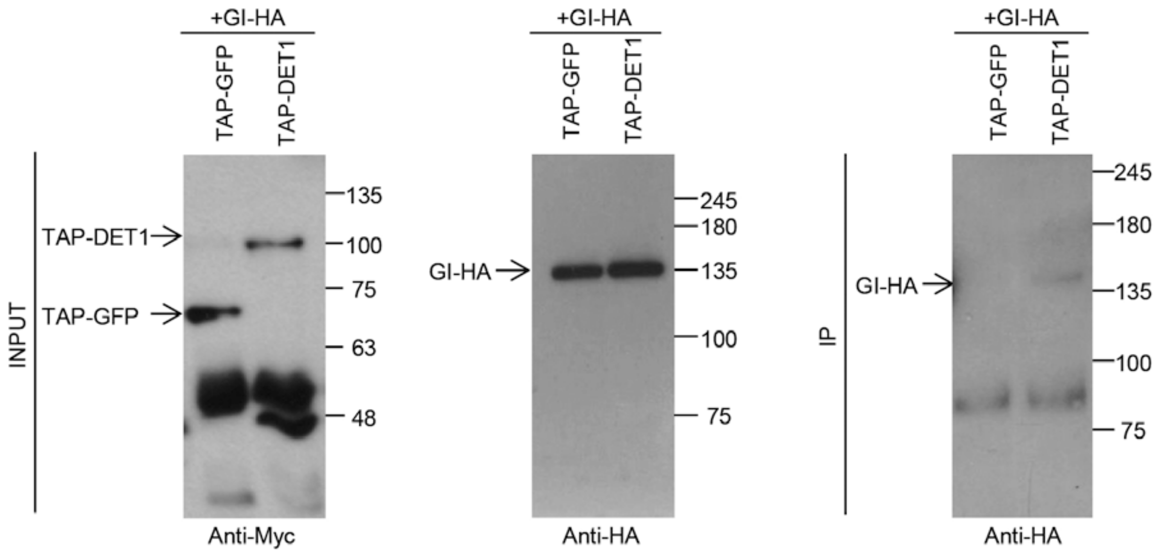

Figure S2 | DET1 directly interacts with GI. Uncropped immunoblot images of Fig. 4c.

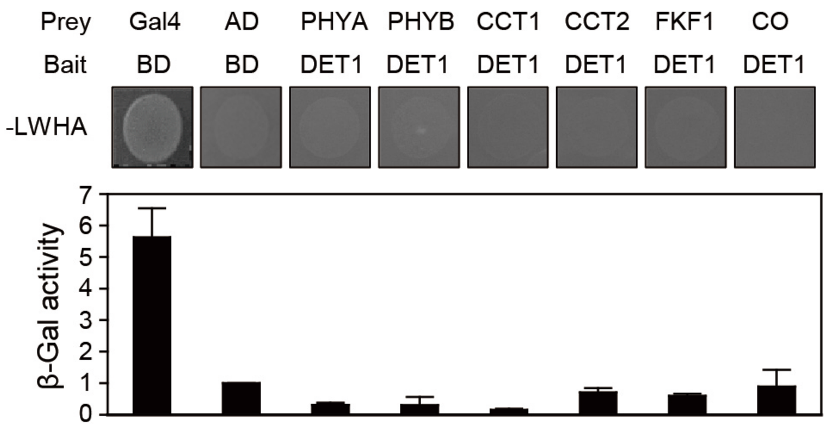

Figure S3 | DET1 does not interact with circadian clock-related proteins in a yeast-2-hybrid assay. DET1 does not interact with PHYA, PHYB, CCT1, CCT2, CO, and FKF1 in yeast-2-hybrid assays. DET1 was used as bait in the pGBK vector. For preys, PHYA, PHYB, CCT1, CCT2, CO, and FKF1 were used. Gal4 indicates a positive control. Empty pGBKT7 (BD) and pGADT7 (AD) vectors were a negative control. SD medium (-LWHA; lacking medium of tryptophan, leucine, histidine, and adenine) was used for selection of the interaction between bait and prey proteins. β-Galactosidase (β-Gal) activity assays were performed according to the manufacturer's protocol. Means and standard deviations were obtained from three biological replicates. This experiment was replicated at least three times with similar results.

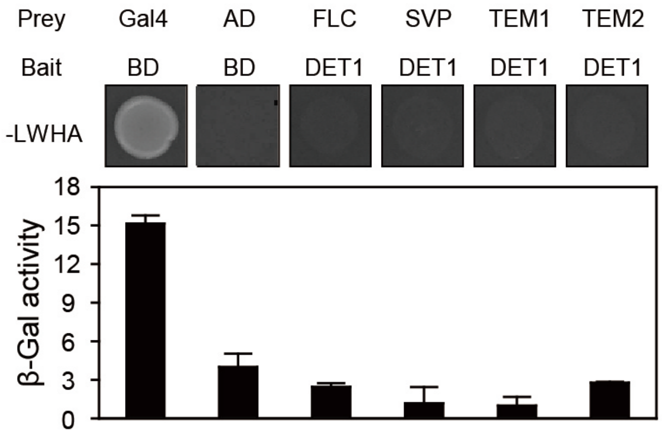

Figure S4 | DET1 does not interact with repressors of *FT* in a yeast-2-hybrid assay. DET1 does not interact with FLC, SVP, TEM1, and TEM2 in yeast-2-hybrid assays. DET1 was used as bait in pGBK vector. For preys, FLC, SVP, TEM1, and TEM2 were used. Gal4 indicates a positive control. Empty pGBKT7 (BD) and pGADT7 (AD) vectors were a negative control. SD medium (-LWHA; lacking medium of tryptophan, leucine, histidine, and adenine) was used for selection of the interaction between bait and prey proteins. β-Galactosidase (β-Gal) activity assays were performed according to the manufacturer's protocol. Means and standard deviations were obtained from three biological replicates.

Table S1. Effect of *det1* mutation on flowering time in different mutant backgrounds.

Genotype	Rosette leaves at bolting LD (16L:8D)	Rosette leaves at bolting SD (10L:14D)
Wild type (Col-0)	12.1 ± 0.9	37.7 ± 3.3
det1-1	9.0 ± 1.3	12.9 ± 2.2
cry2-1	19.4 ± 2.4	42.1 ± 7.8
cry2-1 det1-1	11.4 ± 0.9	17.9 ± 3.5
gi-1	35.5 ± 1.8	52.2 ± 4.0
gi-1 det1-1	29.9 ± 1.7	40.3 ± 4.4
fkf1-t	20.1 ± 1.0	43.0 ± 4.4
fkf1-t det1-1	12.6 ± 1.1	24.3 ± 2.2
co-101	31.8 ± 2.2	48.8 ± 3.6
co-101 det1-1	21.8 ± 1.6	25.6 ± 4.9
ft-1	31.7 ± 1.2	51.7 ± 4.3
ft-1 det1-1	29.0 ± 1.5	40.3 ± 3.0
soc1-2	18.2 ± 1.1	56.2 ± 2.9
soc1-2 det1-1	14.0 ± 2.1	27.0 ± 2.6

Genotype	Rosette leaves at bolting LD (16L:8D)	Rosette leaves at bolting SD (8L:16D)
Wild type(Col-0)	11.8 ± 0.8	44.9 ± 3.7
det1-1	10.4 ± 0.5	16.8 ± 1.0
ft-1	40.8 ± 3.8	69.0 ± 3.9
soc1-2	20.6 ± 1.5	63.1 ± 2.9
ft-1 soc1-2	48.4 ± 4.5	79.8 ± 5.3
ft-1 soc1-2 det1-1	52.4 ± 3.6	85.9 ± 6.6

Table S2. Primer sequences used in this study.

Construct	Primer	Sequence (5’ → 3’)
**Real-time PCR**		
ACT2	ACT_F	TGGGATGAACCAGAAGGATG
	ACT_R	AAGAATACCTCTCTTGGATTGTGC
CO	CO_F	GCCTACTTGTGCATGAGCTG
	CO_R	GTTTATGGCGGGAAGCAAC
FT	FT_F	GGTGGAGAAGACCTCAGGAA
	FT_R	GGTTGCTAGGACTTGGAACATC
FKF1	FKF1_F	GTTGTACCGCCTCCAAGACT
	FKF1_R	AGATGATGACCCTACCACACG
GI	GI_F	TGCATCTGGTGTAAGGCTACC
	GI_R	CCTATAGCCCGCAAGAAGTG
SOC1	SOC1_F	AACAACTCGAAGCTTCTAAACGTAA
	SOC1_R	CCTCGATTGAGCATGTTCCT
FLC	FLC_F	GCTACTTGAACTTGTGGATAGCAA
	FLC_R	GGAGAGGGCAGTCTCAAGGT
***FT* promoters**		
I region	pFT_I_F	CTGCGACTGCGACCTATTTT
	pFT_I_R	GCCACTGTTCTACACGTCCA
II region	pFT_II_F	ACTTGGCGGTACCCTACTT
	pFT_II_R	ATATCTCCCACTTGGTAG
III region	pFT_III_F	GTCGAGAGAGGTATCTTGTTAAAG
	pFT_III_R	ATCATAGGCATGAACCCTCTACAC
IV region	pFT_IV_F	TATGTGTAGAGGGTTCATGCCTATG
	pFT_IV_R	TGGCCATAACCTTTAGAGTG
V region	pFT_V_F	CCAAGAGTTGAGATTGGTGGA

	pFT_V_R	CCAAGAGTTGAGATTGGTGGA
VI region	pFT_VI_F	TCCACCAACTTCTTGCATAA
	pFT_VI_R	CCACAACAGAAATTCATCAA
UBI10	UBI10_F	TTGCCAATTTTCAGCTCCAC
	UBI10_R	TGACTCGTCGACAACCACAA
***FLC* promoters**		
I region	pFLC_I_F	TGTCCACACATATGGCAATAGCTCAA
	pFLC_I_R	CAAGCTGATACAAGCATTTCACCAA
II region	pFLC_II_F	CCTAATTTGATCCTCAGGTTTGGG
	pFLC_II_R	CCGACGAAGAAAAAGTAGATAGGCAC
III region	pFLC_III_F	GTCATTCACGATTTGTTTGATACGATCTG
	pFLC_III_R	GATCTCCCGTAAGTGCATTGCA
FUS3	FUS3_F	ATGTGGCACGTGGGAAATAG
	FUS3_R	GTGGCAAGTGTTGATCATGG
